# Supplementary material for: Experimental and DFT Study of Monensinate and Salinomycinate Complexes Containing {Fe3(µ3–O)}7+ Core
Source: Molecules. 2024 Jan 11;29(2):364. doi: 10.3390/molecules29020364 (PMC10818969; doi:10.3390/molecules29020364)
Supplement: Supplementary file 1 [file molecules-29-00364-s001.zip › molecules-2810336-supplementary.pdf]

molecules

MDPI

Article

Experimental and DFT Study of Monensinate and Salinomycinate Complexes Containing $\{Fe_3(\mu_3–O)\}^{7+}$ Core

Nikolay Petkov [1,*], Alia Tadjer [1], Elzhana Encheva [1,2], Zara Cherkezova-Zheleva [3], Daniela Paneva [3], Radostina Stoyanova [4], Rositsa Kukeva [4], Petar Dorkov [5] and Ivayla Pantcheva [1,*]

[1] Faculty of Chemistry and Pharmacy, Sofia University “St. Kliment Ohridski, 1164 Sofia, Bulgaria; tadjer@chem.uni-sofia.bg (A.T.), eencheva@ipc.bas.bg (E.E.)
[2] Institute of Physical Chemistry, Bulgarian Academy of Sciences, 1113 Sofia, Bulgaria; eencheva@ipc.bas.bg
[3] Institute of Catalysis, Bulgarian Academy of Sciences, 1113 Sofia, Bulgaria; zzhel@ic.bas.bg (Z.C-Z.), daniela@ic.bas.bg (D.P.)
[4] Institute of General and Inorganic Chemistry, Bulgarian Academy of Sciences, 1113 Sofia, Bulgaria; radstoy@svr.igic.bas.bg (R.S.); rositsakukeva@yahoo.com (R.K.)
[5] Research and Development Department, Biovet Ltd., 4550 Peshtera, Bulgaria; p_dorkov@biovet.com
* Correspondence: ahnp@chem.uni-sofia.bg (N.P.); ipancheva@chem.uni-sofia.bg (I.P.); Tel.: +359-2-8161446 (N.P., I.P.)

Supplementary information

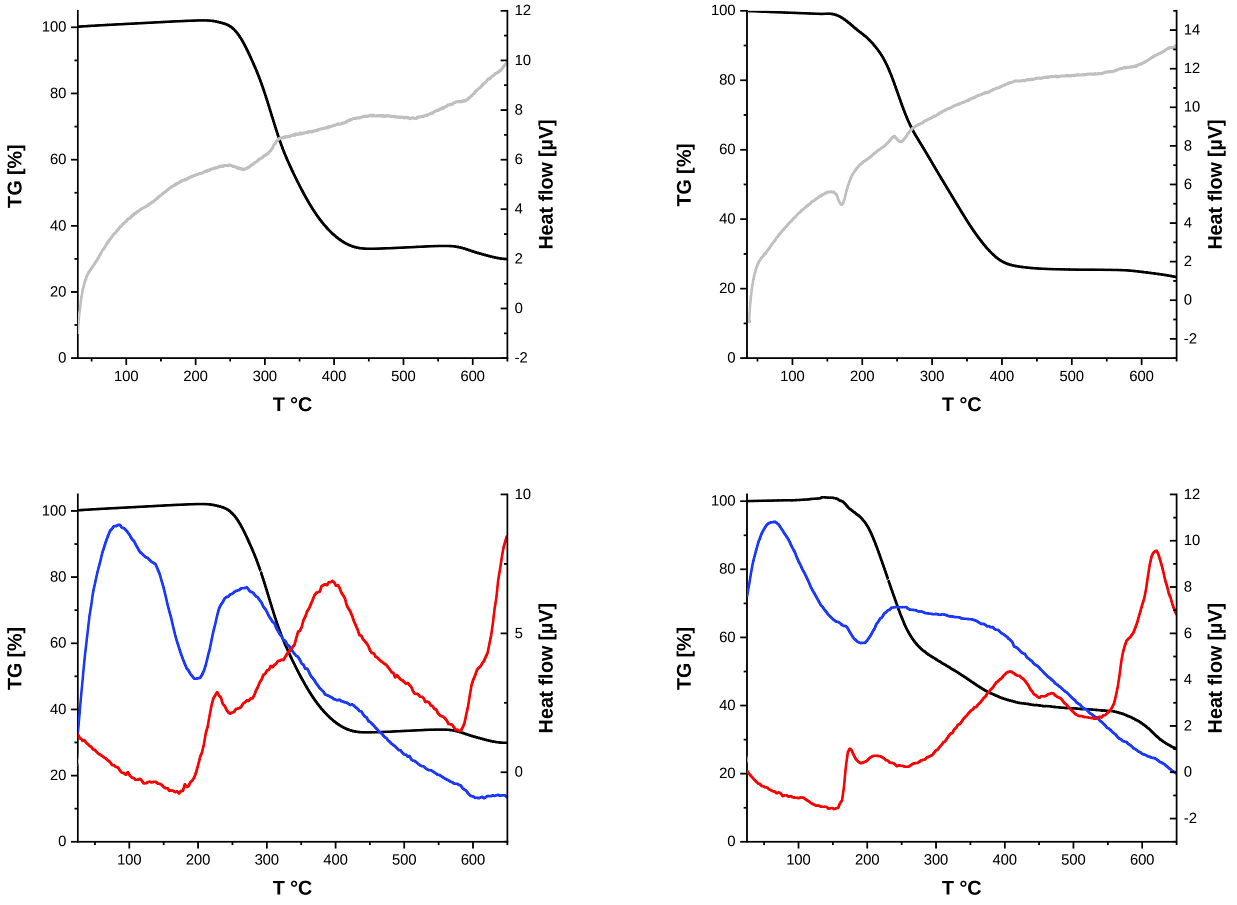

Figure S1. TGV–DTA (top) and TGV–MS (bottom) curves of MonH / complex **1** (left); SalH / complex **2** (right). Colour code: weight decrease (black); endo- and exothermic effects (grey); H_2O loss (blue); CO_2 loss (red).

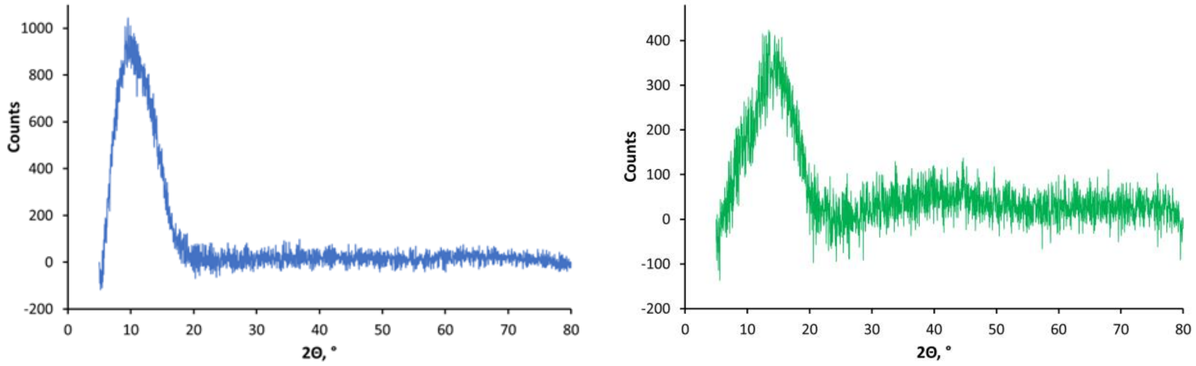

(a) (b)

Figure S2. Powder XRD diffraction of (a) complex **1** and (b) complex **2**.

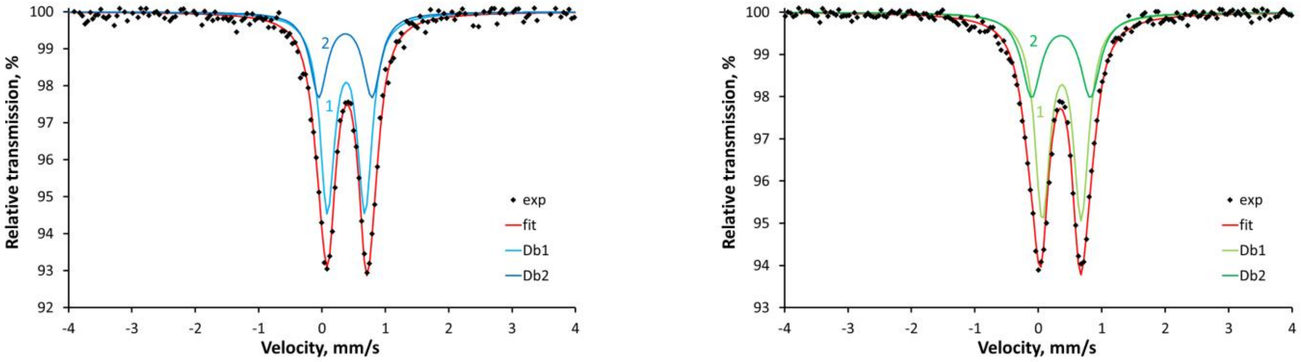

(a) (b)

Figure S3. ^{57}Fe Mössbauer spectra of (a) **1** and (b) **2** at 77 K.

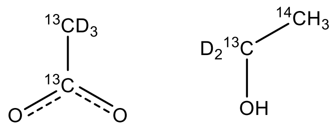

Figure S4. Isotopic substitution of selected carbons and hydrogens in structure **B**.
